# Oral *Helicobacter pylori*: a scoping review and pilot study on the PCR-culture detection paradox

**DOI:** 10.1007/s00784-026-07134-w

**Published:** 2026-09-17

**Authors:** Erin Miller, Jasmin Pyu Pyu Wai Htun, Julie-Anne Martis, Kausar Sadia Fakhruddin, Eng Chua, Alfred Tay, Hooi Ee, Erwin A. Paz, Victor Haruo Matsubara

**Affiliations:** 1 UWA Dental School, The University of Western Australia, 17 Monash Avenue, Nedlands 6009 Perth, WA Australia; 2 The Marshall Centre for Infectious Diseases Research and Training, Helicobacter Research Laboratory, Perth, WA Australia; 3 Department of Gastroenterology, Sir Charles Gairdner Hospital, Perth, WA Australia

**Keywords:** Oral cavity, Nested PCR, Bacterial culture, Dental plaque, Oral mucosa

## Abstract

**Objective:**

The role of the oral cavity as a reservoir for *Helicobacter pylori* remains controversial. This hybrid study, combining a scoping review and a pilot clinical study, aimed to synthesise global evidence on oral *H. pylori* detection and investigate the discrepancy between molecular and culture-based findings.

**Materials and methods:**

A scoping review following JBI and PRISMA-ScR guidelines identified human studies reporting oral *H. pylori* detection using molecular and/or culture-based methods. Review findings guided a pilot clinical study in which supragingival plaque and oral mucosal swabs were collected from symptomatic and asymptomatic *H. pylori*-positive individuals. Detection was performed using nested PCR (16 S rRNA and *ureA*) and selective microaerophilic culture.

**Results:**

Thirty-nine studies involving over 3,000 participants were included. Molecular detection rates varied widely (0–90%), while culture-confirmed viable *H. pylori* was exceedingly rare and restricted to protected anaerobic oral niches. In the pilot study (*n* = 8), PCR detected *H. pylori* DNA in 50% of participants, with higher detection in mucosal swabs (37.5%) than supragingival plaque (25%). No viable organisms were recovered by culture, which was dominated by commensal oral species.

**Conclusion:**

Both the scoping review and pilot study consistently demonstrated a PCR-positive/culture-negative pattern for oral *H. pylori*. These findings suggest that oral detection likely reflects transient bacterial presence rather than stable colonisation. Future studies should employ multi-gene molecular assays, targeted sampling, viability-based techniques, and gastric–oral strain comparisons to clarify the biological significance of oral *H. pylori* detection.

**Clinical Relevance:**

Although *H. pylori* DNA is frequently detected in oral samples, viable organisms are rarely identified. Oral PCR positivity should therefore not be interpreted as active infection, and clinical management should continue to rely on established gastric diagnostic pathways.

**Supplementary Information:**

The online version contains supplementary material available at https://doi.org/10.1007/s00784-026-07134-w.

## Introduction

*Helicobacter pylori* is a gram-negative, microaerophilic bacterium estimated to be present in 50% of the global population and well known for causing gastritis, gastric/duodenal ulcers, and stomach cancer [1, 2]. Oral manifestations reported in association with *H. pylori* infection include halitosis, recurrent aphthous stomatitis, and burning sensations of the tongue; however, these findings are non-specific, and their relationship with the presence of *H. pylori* within the oral cavity remains uncertain [3]. These manifestations are generally linked with vitamin deficiency conditions caused by *H. pylori*-positive gastric disease [4]. Systemic antibiotic therapies have proven successful in treating gastric *H. pylori* infection [5]. Despite this, the reinfection rate remains high [5], prompting research into potential extra-gastric reservoirs. The oral cavity has emerged as a site of significant interest, as it could represent a major route of transmission if *H. pylori* successfully colonises oral microenvironments, potentially serving as a risk factor for gastric reinfection following eradication therapy [6, 7].

However, evidence for the oral cavity as a true reservoir remains contentious. A critical barrier to resolving this debate is the fundamental discordance between detection methodologies. On one hand, molecular techniques, particularly polymerase chain reaction (PCR), consistently report a high prevalence of *H. pylori* DNA in oral samples [8, 9]. On the other hand, traditional culture, the historical gold standard for confirming viable, metabolically active bacteria, frequently yields negative results [10, 11]. This persistent PCR-positive/culture-negative paradox forces a central question: does the oral cavity represent a genuine reservoir for colonizing, viable *H. pylori*, or does it merely contain transient DNA fragments and non-viable cells regurgitated from the stomach?

Therefore, this study employed a dual-method design to directly address this methodological gap and empirically test these competing hypotheses. First, we conducted a systematic scoping review to comprehensively map and synthesize the global evidence on *H. pylori* detection in the oral cavity, with a specific focus on quantifying the disparity between PCR-based and culture-based results. Second, informed by the inconsistencies identified in the review, we performed a pilot clinical study to empirically test this discordance under standardized conditions. The primary aim of the pilot component was to directly compare highly sensitive nested PCR with culture for detecting *H. pylori* in matched dental plaque and oral mucosa samples from a single cohort. Through this integrated approach, we seek to clarify whether the prevalent PCR-positive/culture-negative phenomenon reflects a true biological state or a methodological limitation, with the ultimate goal of informing the design of a larger, definitive study to conclusively determine the oral cavity’s role as a reservoir for *H. pylori*.

## Materials and methods

### Overall study design

To address the pervasive contradictions in the literature regarding the detection of oral *H. pylori*, this study adopted a novel hybrid methodology. This approach synergistically combined evidence synthesis with primary data collection through two integrated components:

- A systematic scoping review was conducted to comprehensively map the existing global evidence, quantify the reported prevalence rates of *H. pylori* detection via different methods (PCR vs. culture), and identify the central methodological challenges and knowledge gaps.
- Guided by the findings of the scoping review, a pilot clinical study was then conducted. Its specific aim was to serve as a controlled, empirical test of the primary discrepancy identified in the literature, the discordance between PCR-based and culture-based detection.

The rationale for this design was to ensure that the clinical pilot study was not only an isolated investigation but also a targeted validation of a well-defined, field-wide problem. This creates a robust feedback loop where the review informs the trial’s design, and the trial’s results validate the trends observed in the review. The pilot clinical study is reported according to the STROBE (Strengthening the Reporting of Observational Studies in Epidemiology) guidelines for observational studies [12]. 

### Scoping review

#### Framework and eligibility criteria

The scoping review was conducted in accordance with the Joanna Briggs Institute (JBI) methodology for scoping reviews [13] and is reported following the Preferred Reporting Items for Systematic reviews and Meta-Analyses extension for Scoping Reviews (PRISMA-ScR) guidelines [14].

The review structure was guided by the Population-Concept-Context (PCC) framework:

- Population (P): Human participants of any age or health status.
- Concept (C): Detection of Helicobacter pylori in the oral cavity.
- Context (C): All original clinical studies employing molecular, for instance, PCR, qPCR, NGS, and/or culture-based detection methods.

*Inclusion criteria* encompassed original human studies (randomized controlled trials, cohort studies, case-control studies, case series ≥ 5 participants) that quantitatively reported the detection of *H. pylori* from any oral site (e.g., dental plaque, saliva, oral mucosa). No restrictions were placed on language or publication status.

*Exclusion criteria* were: (i) animal or in vitro studies; (ii) reviews, editorials, conference abstracts without full data; (iii) studies where oral detection data was not separable from other samples (for instance, pooled gastric and oral data); and (iv) studies not reporting primary detection data (e.g., those focusing solely on genotyping without prevalence).

#### Search strategy

A systematic search was executed across four electronic databases: PubMed/MEDLINE, Scopus, Embase, and Web of Science Core Collection. The search strategy was designed to capture all relevant studies on oral *H. pylori* detection. Separate date filters were applied to reflect the evolving sensitivity of molecular techniques and to provide a contemporary overview of culture attempts:

- For culture-based studies: January 2000 -March 2025.
- For molecular studies: January 2011-March 2025.

The search strategy utilized a combination of Medical Subject Headings (MeSH) and free-text keywords related to three concepts: “Helicobacter pylori”, “Oral Cavity”, and “Detection”. Key search terms included:

- ("*Helicobacter pylori*" OR "*H. pylori*")
- ("oral" OR "mouth" OR "saliva" OR "dental plaque" OR "subgingival" OR "oral mucosa" OR "tongue")
- ("detection" OR "PCR" OR "polymerase chain reaction" OR "culture" OR "isolation" OR "prevalence" OR "molecular")

The full search strategy for PubMed is provided as a supplement (Table S1) and was adapted for the syntax of the other databases. Reference lists of included studies and relevant review articles were also manually screened to identify any additional eligible publications.

#### Study selection and data extraction

The study selection process was performed by two independent reviewers (VHM and KSF). All identified records were imported into EndNote v.10, where duplicates were removed automatically and manually. The reviewers first screened titles and abstracts against the eligibility criteria. The full texts of potentially relevant studies were then retrieved and assessed in detail. Any discrepancies at either stage were resolved through discussion or by consultation with a third reviewer (EP). Data from included studies were extracted into a standardized, piloted data-charting form in Microsoft Excel.

#### Data synthesis and presentation

Given the nature of a scoping review, the objective was to map the available evidence rather than appraise study quality or conduct a meta-analysis. Therefore, the results are presented in two comprehensive summary tables (Table 1: Molecular epidemiological studies for *H. pylori* detection in the oral cavity and Table 2: Studies attempting to culture viable *H. pylori* from the oral cavity).

**Table 1:** Detection of *Helicobacter pylori* in the oral cavity: a summary of molecular epidemiological studies

Study (Year) country	Participant population sample size (*N*)	Oral sample type	PCR method target gene	*H. pylori *positive (*n*; %)	Associations	Co-detection with gastric *H. pylori*	Notes/ limitations
Yamakido et al. 2025 Japan	Older adults (≥ 65 yrs) *N* = 98	Tongue surface swab	qPCR *ureA*	7; 7.1%	Significant association with higher plaque scores (*p* = 0.04). No sig. association with deep pockets	NA	Did not sample subgingival plaque
Muharemovic et al. 2025 Bosnia and Herzegovina	Patients with oral ulcerations vs. Healthy controls. *N* = 80	Cytological smear from the lesion (in ulcer patients) and from the palate, cheek, and tongue (in healthy controls)	qPCR Kit used: Sacace *H. pylori* REAL-TM	10 (5 in Experimental group, 5 in Control group); 12.5% (10/80)	No statistically significant difference. Concludes *H. pylori* is not a risk factor for oral ulcerations and is likely a transient pathogen on the oral mucosa	NA	Sampled multiple oral sites with a specific clinical condition (ulcerations)
Elger et al. 2024 Germany	Children (1–12 years) with S-ECC and endodontically infected deciduous teeth. *N* = 170 teeth from 54 children.	Supragingival plaque and root canal content (from broken teeth)	Conventional PCR *vacA* (s1/s2 and m1/m2 allele regions)	RCT: 32 teeth Plaque: 5 teeth RCT: 18.8% (32/170) Plaque: 2.9% (5/170)	PCR detected *H. pylori* DNA in a significant number of root canals and a few plaque samples.	NA	PCR detected DNA in 37 samples, but viable bacteria were cultured from only 28 of those samples.
Urrutia-Baca et al. 2023 Mexico	36 Families (parents and children); No history of GI disease. *N* = 99	Supragingival and subgingival dental plaque	qPCR *vacA* (s1/m1 region) and *cagA* genes	42; 42.4% (42/99)	Mothers had the highest prevalence (16.2% of the total sample) and the most diverse genotypes. Suggests mother-to-child is a key transmission route	NA	Provides strong evidence for the ‘oral reservoir’ hypothesis in the context of family transmission
Sekar et al. 2023 India	Patients with gingivitis/periodontitis (*n* = 30); oral cancer (*n* = 30); gastric cancer (*n* = 30) *N* = 90	Subgingival plaque	RT-PCR 16 S rRNA (439 bp)	*Group A*: 23; 76.7% *Group B*: 24; 80% *Group C*: 27; 90%	High prevalence across all groups; No significant association between *H. pylori* and oral cancer risk. Strong association with gastric cancer (adjusted RRR = 2.93).	All gastric cancer patients had gastritis.	Cross-sectional design; no direct gastric sample comparison; small sample size per group.
Sruthi et al. 2023 India	Children aged 3–6 years with severe dental caries (ICDAS II codes 5 or 6) *N* = 20	Carious dentine	RT-PCR 16 S rRNA	14; 70%	*H. pylori* associated with higher dmft scores (*p*=0.001) and dt scores (*p*=0.041); More prevalent in ICDAS code 6 lesions.	NA	Small sample size; No gastric samples for comparison; Cross-sectional design
Jara et al., 2022 Chile	Adult patients with gastric symptoms requiring endoscopy; healthy controls Study: 41; Control: 21 *N* = 62	Oral mucosa (OM), Saliva	Nested PCR *cqg42-1* gene (181 bp amplicon)	OM: 12; 29.3% Saliva: 14; 36.8% OM (Control): 12; 57.1%	Strong concordance between OM PCR and gastric RUT (κ = 0.93); No concordance between saliva PCR and RUT	OM PCR showed 100% concordance with gastric RUT	Small sample size
Šeligová et al. 2020 Slovakia	Patients undergoing endoscopy *N* = 81	Saliva	Nested PCR 16 S rRNA	8; 9.9%	Detection limit: ~10³ cells/mL saliva. Three patients had *H. pylori* only in saliva (not detected by gastric tests).	Only 2 patients had identical strains in the stomach and saliva	Low abundance in saliva; sampling variability
Flores-Treviño et al. 2019 Mexico	Patients with periodontal disease *N* = 38	Subgingival dental plaque	qPCR 16 S rRNA (for detection), *vacA* and *cagA* (for genotyping)	23; 60.5%	A significant relationship was found between periodontal status and oral *H. pylori* infection (*P* ≤ 0.05).	NA	Small sample size. No gastric samples to confirm co-infection. cross-sectional study, Uses SYBR Green qPCR; primers were designed in-house
Hamada et al. 2019 Japan	Patients referred for tooth extraction due to dental caries or periodontitis *N* = 87	Saliva and Extracted Teeth (dental pulp)	Nested PCR *ureA* (Urease A)	16 (Total); 8 in saliva; 13 in teeth; 5 in both; 18.4% (16/87)	Significantly higher detection of *H. pylori* in saliva from overweight subjects (BMI ≥ 25) vs. non-overweight (28.6% vs. 5.5%, *P* < 0.05). No significant association with periodontal pocket depth or a clinical history of digestive diseases.	NA	Small sample size; Retrospective study; Semi-quantitative (CFU ranges estimated from nested PCR).
Kadota et al. 2019 Japan	A 29-year-old female patient with duodenal ulcers and confirmed gastric *H. pylori* infection *N* = 1	Supragingival & subgingival plaque (from 6 regions) and Saliva	Nested PCR *ureA* (Urease A)	Positive in 3/6 dental plaque regions and saliva before therapy.	*H. pylori* was present in the oral cavity before gastric eradication therapy.	The patient had a confirmed gastric *H. pylori* infection	Case report: results are not generalizable. Uses a highly sensitive nested PCR
Zhao et al. 2019 China	Patients diagnosed with chronic non-atrophic gastritis. *N* = 80	Tongue Coating (collected by scraping the dorsum)	16 S rRNA (NGS, Illumina MiSeq platform) V3-V4 hypervariable regions	48 (*H. pylori*+ group total: 35 *CagA* + + 13 *CagA-*); 60% (48/80)	*H. pylori*+/CagA+ infection significantly reduced gastric bacterial diversity & composition. The tongue microbiota composition was not significantly altered by *H. pylori* status.	Samples were from patients with confirmed gastric *H. pylori* status	The study identifies associations, not causation.
Ansari et al. 2018 Pakistan	Subjects with periodontal infections. 278 with gastric complications (cases) and 289 with normal gastric mucosa (controls) *N* = 567	Dental plaque	Conventional PCR 16SRNA, 16 S rDNA *babA*,* cagA*,* ureA*,* ureC*,* vacA*.	247 (based on 16SRNA detection across all subjects); 43.6% (247/567)	No significant overall association was found between oral *H. pylori* and gastric infections/complications	Gastric biopsy of all participants & analyzed by PCR for *H. pylori*.	The study only included subjects with periodontal infections, limiting generalizability
Nomura et al. 2018 Japan	Children and adolescents (aged 1–19 years) with inflamed dental pulp due to ECC or trauma *N* = 131 (inflamed pulp specimens)	Inflamed dental pulp tissue	Nested PCR *ureA*	51; 38.9%	*H. pylori* was more frequently detected in primary teeth than in permanent teeth. *H. pylori* strains demonstrated the ability to adhere to human dental pulp fibroblast cells (HDPFs) *in vitro.*	NA	The nested PCR method was highly sensitive, with a detection limit of 1–10 cells, even from inflamed pulp specimens
Wongphutom et al. 2018 Thailand	Asymptomatic, healthy adults (aged 18–60) *N* = 110	Both saliva and stool	Semi-nested PCR *vacA* (semi-nested PCR), 16 S rRNA (real-time PCR)	70 (in saliva); 63.6% (70/110)	The overall prevalence of *H. pylori* in asymptomatic individuals was high (64%)	NA	No significant association between *H. pylori* colonization and age, gender, occupation, or brushing frequency.
Aksit Bicak et al. 2017 Turkey	Children aged 5–16 years with and without dyspepsia *N* = 100 (70 dyspeptic, 30 control)	Supragingival dental biofilm and unstimulated saliva	RT-PCR 16 S rRNA and 23 S rRNA	*Dental Biofilm* (both genes +): 53/100 (53%) *Dyspeptic Gastric Hp* (+): 45/58 (77.6%) *Dyspeptic Gastric Hp* (-): 8/12 (66.7%) **Control Group**: 5/30 (16.7%) *Saliva (both genes +)*: 65/100 (65%) *Dyspeptic Gastric Hp (+)*: 44/58 (75.9%) *Dyspeptic Gastric Hp (-)*: 9/12 (75.0%) *Control Group*: 12/30 (40.0%)	Detection rates in dental biofilm and saliva were significantly higher in dyspeptic children than in controls The quantity of *H. pylori* in dental biofilm was significantly higher in the gastric *H. pylori*-positive group than in the negative and control groups. Oral *H. pylori* presence (both genes positive) was associated with higher halitosis (BANA test), higher DMFS index, and lower salivary pH.	*H. pylori* was also frequently found in the oral cavity of children without gastric infection, indicating it can exist independently.	Simultaneous amplification of two genes was used for more reliable detection
Castro-Munoz et al. 2017 Mexico	Asymptomatic children under 5 years *N* = 162	Oral swab (rotated around the inside of the cheeks)	Conventional PCR 16 S rRNA and *glmM*	21; 13% (21/162)	The *16 S rRNA* gene was more frequently detected (21 samples) than the *glmM* gene (9 samples).	NA	Using multiple gene targets improves detection sensitivity, given strain diversity
Medina et al. 2017 Argentina	Adult dyspeptic patients (18–80 years) undergoing endoscopy *N* = 61	Dental plaque and saliva	Conventional PCR For detection: *ureA* For genotyping: *cagA*,* babA2*,* vacA*	31; 50.8% (31/61) in both oral and gastric samples	Genotype concordance between oral and gastric samples from the same patient was found in 38.7% (12/31) of cases.	All 61 patients provided both oral and gastric samples. Only 38.7% had genotype concordance	No statistical correlation was found between the virulence genes (*cagA*, *babA2*) and specific gastroduodenal or oral diseases.
Mendoza-Cantú et al. 2017 Mexico	Children (3–16 years) without dyspeptic symptoms *N* = 100	Supragingival dental plaque	qPCR Nested PCR 16 S rRNA For genotyping: *cagA*, *vacA* (s1/s2, m1/m2 alleles)	38; 38% (38/100)	A significant association was found between oral *H. pylori* infection and gingival status (*P* < 0.001).	NA	The study focused solely on asymptomatic children, and no gastric samples were collected.
Valadan et al. 2017 Iran	50 patients with chronic periodontitis and 50 controls *N* = 100	Supragingival plaque (both groups) and subgingival plaque (periodontitis group only)	Conventional PCR 16 S rRNA, *ureC (glmM)* For genotyping: *cagA*,* vacA* (s1/s2, m1/m2), *babA2*	5; 5% (5/100)	No significant correlation was found between any *H. pylori* genotype and chronic periodontitis	NA	The low number of positive cases (*n* = 5), limits the power to find significant associations with clinical conditions.
Urban et al. 2017 Poland	Adults with a positive gastric urease test *N* = 108	Saliva and dental plaque (combined sample)	Conventional & Nested PCR Multiple: 16 S rRNA (two primer sets), *UreA*, 860 bp DNA region, *cagA*	50; 46% (50/108)	*H. pylori* in the oral cavity is associated with poorer oral hygiene.	All 108 participants were gastric *H. pylori* positive.	Positive result defined by detection of at least 2 different target genes to minimize false positives/negatives.
Ismail et al. 2016 United Kingdom	Adults undergoing upper GI endoscopy *N* = 49	Dental Plaque	Single-step PCR Nested PCR 860 bp fragment of *H. pylori* genomic DNA (primers: EHC-U/EHC-L, then nested with EN3-U/EN3-L)	**Nested PCR**: 20 (40.8%) before endoscopy, 21 (42.8%) after endoscopy **Single-step PCR**: 0	83% (15/18) of gastric Hp+ patients (by histology) were also oral Hp + by nested PCR. Suggests a correlation between oral and gastric infection.	Of 18 gastric Hp+ patients (by histology), 15 (83.3%) were also positive in dental plaque (by nested PCR).	Nested PCR detected *H. pylori* in some patients negative by gastric histology, suggesting higher sensitivity or detection of coccoid forms.
Kazanowska-Dygdała et al. 2016 Poland	Patients with oral leukoplakia (L) and oral lichen planus (OLP) *N* = 166 (54 L + 72 OLP + 40 Control)	Plaque from premolar/molar interdental spaces or periodontal pockets	Nested PCR 860 bp fragment of *H. pylori* genomic DNA	L Group: 11; 20.4% OLP Group: 17; 23.6% Control Group: 0% Overall, in Pathology Groups: 28; 22.2%	*H. pylori* DNA was significantly associated with (L and OLP) lesions compared to healthy controls. Worse periodontal status (higher API, BOP, PD) was observed in the pathology groups and was more pronounced in *H. pylori-*positive patients within these groups.	NA	Lack of concurrent gastric *H. pylori* status, so the origin of the oral bacteria is unknown
Amiri et al. 2015 Iran	Patients with chronic periodontal diseases *N* = 45	Dental Plaque (from at least one anterior and one posterior tooth)	Conventional Single-step PCR & LAMP assay *ureC (glmM)*	20; 44%	A more sensitive method (LAMP) detected *H. pylori* in 66.67% of samples. Combined, either PCR or LAMP was positive in 77.78% of samples, suggesting a high oral prevalence that may be underestimated by PCR alone.	NA	The study’s main aim was to compare a new LAMP assay against PCR, not to correlate with gastric infection.
Ogaya et al. (2015) Japan	Children & adolescents (2–19 yrs) with inflamed dental pulp *N* = 40 Children (3–8 yrs) *N* = 40	Inflamed dental pulp Saliva	Conventional PCR *ureA*	6; 15%	The infected root canal may be a reservoir for *H. pylori*. The PCR system was highly specific and sensitive (1–10 CFU/reaction). Saliva was not a reliable sample for detecting *H. pylori* with this method	NA	*H. pylori* was detected in the inflamed pulp but not in saliva. Pulp and saliva samples were from different groups of individuals. Small sample size.
Abadi et al. (2014) Iran	Dyspeptic patients admitted for gastroscopy Not explicitly stated for the total cohort (Data presented per symptom group)	Dental Plaque	Conventional PCR *ureC*	Varies by clinical symptom group (e.g., 72.6%; 62/82 for epigastric pain without nocturnal awakening).	PCR detection rate in plaque was consistently high across various dyspeptic symptoms.	Both gastric biopsy and dental plaque were tested from the same patients.	Suggests the oral cavity (dental plaque) is a reservoir that can lead to gastric reinfection post-therapy. The PCR results are presented aggregated by symptom groups.
Bharath et al. 2014 India	Dyspeptic adult patients advised for endoscopy *N* = 56	Supragingival plaque	Real-time PCR *ureA*	19; 33.9%	The same strain of *H. pylori* is found simultaneously in plaque and gastric mucosa.	34/56 (60.7%) gastric biopsies were PCR-positive. In 6 patients, plaque was positive, but the gastric mucosa was negative	Small sample size; no healthy control group
Cai et al. 2014 China	Children (4–15 years) with upper GI symptoms undergoing endoscopy *N* = 46 (patients with confirmed gastric *H. pylori* infection)	Dental plaque and gargle (saliva)	Conventional PCR and TA cloning-based sequencing 16 S rDNA (V1 variable region) and *CagA* gene	26 (56.5%; 26/46) for 16 S rDNA in oral samples 12 (26.1%; 12/46) for *CagA* gene in oral samples)	Suggests distinct *H. pylori* strains exist in the oral cavity, which may not be the main reservoir for gastric infection in children.	All 26 oral-positive patients were also gastric *H. pylori* positive	PCR for 16 S rDNA can cross-react with other oral *Helicobacter* or *Campylobacter* species. Use of the *CagA* gene improved specificity
Wang et al. 2014 China	Symptomatic individuals with stomach pain (*n* = 159) and asymptomatic individuals with no stomach complaints (*n* = 118) *N* = 277 (total recruited)	Saliva and dental plaque	Conventional PCR *GlmM*	Not explicitly stated for the entire cohort	PCR detected *H. pylori* in the oral cavity of individuals who were UBT-negative (no gastric infection).	Yes. Group A (*n* = 159) had both oral (HPS/HPF/PCR positive) and gastric (UBT positive) infections.	PCR results are not presented independently; they are grouped with antigen tests (HPS/HPF)
Hirsch et al. (2012) Germany	Children with S-ECC requiring tooth extraction *N* = 10 teeth from 3 Children	Dental Plaque Root Canal	Conventional PCR 16 S rRNA (Genus-specific *Helicobacter*)	**Plaque**: 40%; 4/10 samples **Root Canal**: 20%; 2/10 samples	PCR detected *H. pylori* DNA in both plaque and root canal samples. However, live bacteria were only cultured from the PCR-positive root canals, not from the PCR-positive plaque.	NA	Small sample size (only 3 patients, 10 teeth). The gastric *H. pylori* status is unknown.
Gao et al. (2011) China	Patients with chronic gastric diseases and Hp infection (confirmed by gastric biopsy) *N* = 96	Dental Plaque	Conventional PCR *ureC* and *cagA*	79; 82.3%	Dental plaque (82.3%) Gargles/Saliva (51.1%) Tongue dorsum (37.5%)	All 96 patients had confirmed gastric Hp infection.	No genotyping to confirm that oral and gastric strains are identical.
Wichelhaus et al. (2011) Germany	Adolescents (mean age 12.7) before/during orthodontic treatment *N* = 11 (93 plaque samples)	Dental Plaque	Nested PCR 860 bp DNA gene (primers EHC-U/EHC-L and ET-SU/ET-SL)	9 (82%) at t1 (baseline)	HP prevalence decreased during orthodontic therapy (82% to 54%).	All 11 patients were consistently negative for gastric HP via the ¹³C-urea breath test at all time points.	Small sample size (*n* = 11). No genotyping. The “860 bp DNA” target is not a standard, well-defined gene

NA: Not Assessed or Not Applicable, *PCR *Polymerase Chain Reaction, *qPCR/RT-PCR* Quantitative/Real-Time PCR, *NGS* Next-Generation Sequencing, *S-ECC* Severe Early Childhood Caries, *RCT* Root Canal Treatment, *RUT* Rapid Urease Test (a common gastric test for *H. pylori*), *UBT* Urea Breath Test (a common gastric test for *H. pylori*), *dmft/DMFS *Decayed, Missing, and Filled Teeth (deciduous/permanent) index, *BANA test* A test used to detect enzymatic activity associated with halitosis, *BMI* Body Mass Index, *LAMP* Loop-Mediated Isothermal Amplification, *CFU* Colony Forming Units, *bp* Base Pairs, *API* Approximal Plaque Index, *BOP* Bleeding on Probing, *PD *Pocket Depth, *RRR* Relative Risk Ratio, *GI* Gastrointestinal

This table summarizes selected studies investigating the presence of *Helicobacter pylori* in the oral cavity using polymerase chain reaction (PCR)-based methods. The data presented include study characteristics, participant demographics, oral sample type, molecular detection targets and methods, prevalence rates, key associations with oral and systemic conditions, and co-detection with gastric infection

**Table 2:** Studies attempting to culture *Helicobacter pylori* from the oral cavity

Study (Year) country	Participant population sample size (*n*)	Oral sample type	Culture medium incubation atmosphere	Confirmation method	*H. pylori *positive (*n*; %)	PCR result from same study	Key findings notes/ limitations
Elger et al. 2024 Germany	Children (1–12 years) with S-ECC and endodontically infected deciduous teeth. *N* = 170 teeth from 54 children.	Supragingival plaque and root canal content	GC agar plates supplemented with horse serum, vitamin mix, and antibiotics (colistin, nystatin, trimethoprim, vancomycin). Microaerobic (using CampyGen sachet)	Morphology (electron microscopy), biochemical (urease test), protein profiling (SDS-PAGE, Western blot for Urease B, VacA, HtrA, CagA, etc.), Functional assays in cell culture (CagA phosphorylation, cell elongation, vacuolization, NF-κB activation).	**Root Canal**: 28 teeth 16.5% (28/170) **Plaque**: 0 teeth (0/170)	**Root Canal**: 18.8% positive by PCR (32/170). **Plaque**: 2.9% positive by PCR (5/170).	The root canal provides a protected,**,** microaerobic environment similar to the stomach Viable *H. pylori* can exist in the oral cavity, but only in a specific, protected niche (the root canal)
Abadi et al. (2014) Iran	Dyspeptic patients admitted for gastroscopy Not explicitly stated	Dental Plaque	Brucella Agar + 7% FCS + 10% Sheep Blood + Antibiotics (Polymyxin B, Trimethoprim, Amphotericin B, Vancomycin) Microaerophilic (Anaerobic jar with gas pack)	Biochemical Tests (Catalase, Oxidase, Urease, Gram stain)	Varies by clinical symptom group, e.g., 41.4% (34/82) for epigastric pain without nocturnal awakening.	Yes. For most symptom groups, the culture-positive samples were a subset of the PCR-positive samples. Culture was less sensitive than PCR.	Live *H. pylori* was successfully cultured from dental plaque, confirming it as a viable reservoir. As with PCR, the total patient cohort size is unclear. The culture method, while successful, appears to be less sensitive than the PCR assay used.
Wang et al. 2014 China	Symptomatic & asymptomatic individuals with & without stomach pain for the PCR. Not explicitly stated for the culture subgroup, but results are provided for 363 tests	Saliva	Trypticase Soy Agar (TSA) + 5% sheep blood Microaerophilic (using a “Campylar” jar with a “Campypack” and water)	Oxidase test, Catalase test, *H. pylori* antigen test, Microscopy (Gram stain)	239 (from a total of 363, 65.8% (239/363) culture tests performed	PCR was positive in the oral samples used for culture. The study used a combination of HPS, HPF, and PCR to define oral infection before culture.	Successfully cultured *H. pylori* from saliva using a pre-treatment method. The method used Urea-HCl treatment of the saliva sample before plating. Urea creates a protective ammonia cloud for *H. pylori*, while HCl eliminates other competing oral bacteria, avoiding the use of antibiotics in the culture medium.
Boyanova et al. 2013 Bulgaria	Subjects with oral and/or gastric diseases. The positive strain was from a 9-year-old child with catarrhal gingivitis and chronic gastritis. *N* = 43	Dental plaque (from the internal surface of lower molar teeth)	Columbia blood agar with 1% Isovitalex and either Dent’s or Degnan’s selective supplement Microaerophilic	Colony morphology, Gram stain, biochemical tests (urease, catalase, oxidase), immunofluorescence with monoclonal antibodies, and PCR for virulence genes.	1; ~2.3% (1/43)	The single cultured strain was confirmed by PCR to be positive for the *ureA*, *cagA*, and *vacA s1 m2* genes.	This is a rare, successful isolation of *H. pylori* from dental plaque. The strain was virulent (*cagA+*,* vacA s1)* and resistant to metronidazole and clarithromycin. This case report demonstrates that viable, culturable *H. pylori* can be present in the oral cavity, but it is a rare event.
Yee et al. 2013 China	Symptomatic individuals had chronic indigestion/stomach pain and underwent endoscopy *N* = 201 (63 symptomatic men, 47 symptomatic women, 36 asymptomatic men, 55 asymptomatic women)	Saliva (collected with a swab from under the tongue) Culture was performed only on the 110 symptomatic patients who underwent endoscopy.	Columbia blood agar plate 5% CO₂ at 37 °C	NS	The results are given as a mean positive rate for the UBT+ symptomatic subgroup: 97.5%. The positive rate for the UBT- symptomatic subgroup was 0.0%.	The study did not use PCR	Culture was highly positive (97.5%) in patients with confirmed gastric infection (UBT+), but completely negative (0%) in symptomatic patients without gastric infection (UBT-).
Hirsch et al. (2012) Germany	Children with S-ECC requiring tooth extraction *N* = 10 teeth from 3 Children	Dental Plaque Root Canal	GC Agar + 10% Horse Serum + Vancomycin, Trimethoprim, Nystatin, Colistin Microaerophilic (using Campygen gas packs)	Colony Morphology FESEM Morphology Urease Test 16 S rRNA Sequencing Western Blot (*UreA/B*,* CagA*,* FlaA*, etc.)	**Root Canal**: 20%; 2/10 samples **Plaque**: 0/10 samples	**Root Canal**: The 2 culture-positive samples were also PCR-positive. **Plaque**: 4 samples were PCR-positive but culture-negative.	Plaque samples, even when PCR-positive, did not yield live *H. pylori* upon culture. This could be due to a transient presence, low numbers, or a non-viable state in plaque.
Teoman et al. 2007 Turkey	Dyspeptic patients undergoing upper GI endoscopy. All patients were periodically healthy with optimal oral hygiene. *N* = 67	Subgingival plaque (from the molar region)	Brain Heart Infusion (BHI) agar with 7% horse blood and *H. pylori*-selective supplement (Oxoid-SR 147E) Microaerobic (5% O₂, 10% CO₂, 85% N₂)	Colony morphology, Gram stain, motility, urease, catalase, and oxidase reactions.	None	19/67 (28.3%) positive by PCR in dental plaque.	*H. pylori* may be present in the oral cavity in a non-culturable, coccoid form. The study provides a stark contrast between culture and PCR results, reinforcing that culture is an ineffective method for detecting *H. pylori* from dental plaque.
Loster et al. 2006 Poland	Patients (aged 25–70 years) with dentures and stomatitis prosthetics *N* = 40	Saliva and supragingival plaques	Agar-horse (Horse blood agar) Not explicitly stated.	The study used RAPD-PCR and specific PCR for *cagA*, *vacA*, and *urease* genes to genetically characterize and compare the cultured *H. pylori* strains from the oral cavity and stomach.	Not provided specifically	The key finding was that the DNA patterns of oral and gastric *H. pylori* strains showed no resemblance, indicating they were different strains.	The culture method was successful in isolating the bacterium. The significant finding is the genetic disparity, which argues against a direct transmission link.
Cześnikiewicz-Guzik et al. 2004 Poland	100 female subjects (mean age range 19.9 to 51.8 years), 41.8% of whom had dyspeptic symptoms. *N* = 100	Saliva and plaque from subgingival pockets	Solid selective, enriched medium for *H. pylori* growth (*H. pylori* agar, Becton Dickinson). Not explicitly stated	Colonies were confirmed using Gram stain (showing gram-negative spiral bacteria) and biochemical tests for urease, catalase, and oxidase. The API-Campy test (BioMerieux) was used for final confirmation.	Saliva: 54; 54% Gingival Pockets: 48.3; 48.3%	The study did not use PCR for detection. Gastric infection was determined by the Urea Breath Test (UBT), and oral presence was determined solely by culture.	Study found a high prevalence of *H. pylori* in the oral cavity via culture, similar to the gastric infection rate (~ 51%). However, there was no statistical correlation between the presence of the bacteria in the oral cavity and a concurrent gastric infection.
Umeda et al. 2003 Japan	Japanese subjects with a history of gastritis/peptic ulcers and healthy controls, attending a university hospital. A subset of *N* = 18 subjects was used for culture.	Dental plaque, tongue plaque, saliva	*H. pylori* selective medium (unspecified brand) Microaerophilic	Colony morphology, catalase test, oxidase test, and confirmation by nested PCR	1 (A “spread colony” was observed and confirmed by PCR, but it could not be isolated.) ~ 5.6% (1/18 subjects)	35.1% (20/57 subjects) were positive by nested PCR in the oral cavity.	The presence of unculturable but viable coccoid forms of *H. pylori*. The culture results starkly contrast with the high detection rate of *H. pylori* DNA via nested PCR.
Young et al. 2001 United Kingdom	Dyspeptic patients (age range 29–63 years) attending for an upper gastrointestinal endoscopy *N* = 5	Supragingival dental plaque	Brain Heart Infusion (BHI) agar with 5% horse blood, both with and without *H. pylori* selective supplement. Microaerophilic	Colony confirmation via Gram stain and production of catalase, urease, and oxidase.	None	3 out of 5 patients (60%) were PCR-positive for *H. pylori* in dental plaque.	Despite being detectable by PCR and visualizable by SEM (showing both rod and coccoid forms), *H. pylori* could not be cultured from any dental plaque sample.

PCR: Polymerase Chain Reaction, *S-ECC* Severe Early Childhood Caries, *GC Agar* Gonococcal Agar (a common base for *H. pylori* culture), *FCS* Fetal Calf Serum, *BHI* Brain Heart Infusion, *UBT* Urea Breath Test, *RAPD-PCR* Random Amplification of Polymorphic DNA PCR (a genotyping method), *SEM* Scanning Electron Microscopy, *SDS-PAGE *Sodium Dodecyl Sulfate–Polyacrylamide Gel Electrophoresis, *NF-κB *Nuclear Factor Kappa-Light-Chain-Enhancer of Activated B Cells (a protein complex involved in immune response), *API* Analytical Profile Index (a commercial biochemical identification system), *NS* Not Specified

This table summarizes studies that attempted to isolate viable *Helicobacter pylori* from the oral cavity using culture techniques. It details the culture conditions, confirmation methods, and success rates, and compares the results with PCR-based detection from the same samples. The data highlight the challenges of culturing oral *H. pylori* and the potential for the oral cavity to act as a reservoir for viable bacteria

The data within these tables are accompanied by a narrative synthesis that summarizes and describes the characteristics of the included studies, the methodologies used, the detection rates, and the central theme of discordance between molecular and culture-based findings. The study selection process is detailed in a PRISMA-ScR flow diagram (Supplementary material - Figure S1).

Consistent with JBI guidance for scoping reviews and the PRISMA-ScR framework, no formal risk-of-bias or methodological quality assessment was conducted.

### Pilot clinical study component

#### Study participant recruitment

Participants were recruited into two groups: (1) symptomatic individuals with confirmed gastric *H. pylori* infection by urea breath test (UBT) who reported active upper gastrointestinal symptoms, such as epigastric pain/dyspepsia/reflux, and (2) asymptomatic individuals with confirmed gastric *H. pylori* infection by UBT but without reported gastrointestinal symptoms (asymptomatic carriers). The study group of symptomatic *H. pylori*-positive participants was recruited on a volunteer basis from patients awaiting upper endoscopic examination at the Sir Charles Gairdner Hospital (SCGH) in Perth, WA, Australia. The control group of asymptomatic *H. pylori*-positive participants was recruited from the public.

*Exclusion criteria* included participants who had been taking proton pump inhibitors during the past two weeks and/or had used any type of antibiotics within four weeks before the sample collections. All participants were screened and examined to collect their medical and social history, clinical dental condition, plaque score, and saliva test results.

#### Sample collection

All oral samples were collected at the Oral Health Centre of Western Australia (Perth; Australia). Supragingival plaque samples were collected by performing an upward scrape against the surface of one posterior and one anterior tooth of each participant using sterile curettes. Subgingival plaque sampling was intentionally omitted in this pilot feasibility study to focus initial validation on the more accessible supragingival niche, which remains commonly sampled in the literature despite its potential limitations. Oral mucosal swab samples were collected by rotating a sterile mouth swab against the buccal mucosa, tongue, floor of the mouth, and soft palate. Plaque and oral mucosal swab samples were placed in 0.5 mL of sterile phosphate-buffered saline (PBS) contained in a sterile microfuge tube. All samples were kept refrigerated until processing.

#### PCR detection of *H. pylori*

Total DNA extraction was performed for each dental plaque and oral mucosal swab sample using DNeasy Blood & Tissue Kit (Qiagen, Clayton, VIC, Australia) according to the manufacturer’s instructions. A nested PCR protocol was used with *H. pylori*-specific primers targeting the *16 S rRNA* and *ureA* genes. The thermocycler was run: 95 °C x 60 s; 40 × (95 °C x 15 s, 60 °C x 15 s, 72 °C x 20 s); 70 °C x 5 min. A 1:50 dilution of the PCR products from the first PCR was then used as the template in the second PCR. PCR products were electrophoresed at 100 V for 45 min on 1% Bionic buffer agarose gel stained with gel red nucleic acid stain and imaged with the BioRad GelDoc XR Plus (Bio-Rad Laboratories).

#### Bacterial culture

To maximize bacterial recovery, samples were first centrifuged at 10,000 × g for 10 min at 4 °C to concentrate bacterial cells. The pellet was resuspended in 100 µL of sterile phosphate-buffered saline, and 100 µL aliquots were spread uniformly onto individual Columbia agar plates containing *H. pylori* selective supplement and antibiotics (vancomycin, trimethoprim, cefsulodin, and amphotericin B). The plates were incubated at 37 °C in a 10% CO₂ environment under microaerophilic conditions for up to 10 days, with daily examination for colony formation. Initial examination occurred at 2–4 days, but plates were maintained for the full incubation period to detect slow-growing *H. pylori* colonies. Suspected *H. pylori* colonies, as well as other bacterial colonies growing on the selective agar plates, were picked and streaked on fresh blood agar plates and incubated for two to four days. Morphological observations of the bacterial colonies were recorded. The non-*H. pylori* colonies were further assessed and identified using whole-genome sequencing. Matrix-Assisted Laser Desorption/Ionization Time-of-Flight (MALDI-TOF MS) was not employed in this study because the primary objective of the pilot study was to evaluate the discrepancy between molecular detection and culture-based recovery of *H. pylori*, rather than to comprehensively characterise all cultivable oral microorganisms; non-*H. pylori* isolates of interest were instead identified by whole-genome sequencing.

Next, pure bacterial colonies from the blood agar plates were again uniformly spread onto fresh blood agar plates and incubated. After 2 to 4 days, pure colonies were Gram-stained and imaged under the microscope, and the following biochemical tests were performed for *H. pylori* species confirmation: urease, catalase, and oxidase.

## Results

### Scoping review findings

#### Study selection and characteristics

Our systematic search identified 1,852 records. After duplicate removal and screening, 39 studies met the inclusion criteria, comprising a global body of evidence from over 15 countries. The evidence base included 28 studies [15–42] that used only molecular methods, 7 using only culture [43–49], and 4 studies employing both methodologies [50–53] (Tables 1 and 2). The reviewed literature encompassed diverse patient populations, with most studies (*n* = 26) focusing on adults, while a substantial subset (*n* = 11) investigated oral *H. pylori* in paediatric or adolescent cohorts. The included studies represented a diverse range of designs, with a total pooled sample size exceeding 3,000 participants.

#### Widespread molecular detection of *H. pylori* DNA

The synthesis of molecular epidemiological studies (Table 1) demonstrated that *H. pylori* DNA is frequently detected in the oral cavity, although reported prevalence rates varied substantially from 0% [35] to 90% [18]. This heterogeneity was strongly influenced by geographic region, patient population, and the molecular methods employed. In particular, the choice of PCR assay had a marked impact on detection rates, although other molecular methods can also be used for the detection of *H. pylori*. Eleven studies [20, 21, 23, 24, 27, 28, 32, 33, 35, 36, 42] that utilized nested PCR consistently reported higher sensitivity, including detection rates of 29.3% in oral mucosa [20] and 38.9% in dental pulp [27]. The superior performance of nested PCR was further underscored by Ismail et al. [36], who detected *H. pylori* in 40.8% of plaque samples using nested PCR, whereas their conventional single-step PCR failed to detect any positives. Across studies, *H. pylori* DNA was identified in various oral niches, with some of the highest detection rates reported in subgingival plaque. For example, Sekar et al. observed rates of 76.7–90% [18], Flores-Treviño et al. reported 60.5% [22], Urrutia-Baca et al. identified 42.4% positivity in supragingival plaque [17]. Notably, several studies reported significant associations between oral *H. pylori* detection and periodontal disease [22], gastric cancer [18], and intrafamilial transmission [17], suggesting that oral carriage may hold clinical relevance.

#### Rare success in cultivating viable *H. pylori*

In contrast to molecular findings, culture-based studies (Table 2) revealed that isolating viable *H. pylori* from the oral cavity is exceedingly rare. Most investigations reported a 0% culture success rate. Even among the small number of studies that achieved positive cultures, yields were very low, generally below 5%. Umeda et al. [48]. reported a culture positivity of approximately 5.6%, while Boyanova et al. documented 2.3% [43].

Successful cultivation appeared to depend on highly specific conditions. These included sampling from structurally protected, microaerophilic niches such as root canals, where reports ranged from 16.5% (Elger et al.) [50] to 20% (Hirsch et al.) [53] In other cases, culture success required specialized pre-treatment protocols designed to suppress competing oral flora, such as those described by Oshowo et al. [52]. Collectively, these findings highlight the severe difficulty of cultivating viable *H. pylori* from oral samples, even when molecular methods confirm the presence of bacterial DNA.

#### The central discrepancy: PCR-positive/culture-negative

Across the scoping review, the most consistent and defining observation was the substantial discordance between molecular and culture-based detection. Numerous studies reported high PCR positivity in samples that were simultaneously culture-negative. Teoman et al. [45] for example, detected *H. pylori* DNA in 28.3% of dental plaque samples, yet were unable to culture any viable organisms. Similarly, Young et al. [49] visualized both rod-shaped and coccoid forms through SEM in PCR-positive plaque specimens but failed to obtain growth in culture.

This pervasive PCR-positive/culture-negative pattern, documented across diverse populations, sampling sites, and methodologies, establishes it as a fundamental and ubiquitous characteristic of oral *H. pylori* research. Together, these findings underscore the unresolved question of whether molecular detection reflects true oral colonization or the presence of non-viable or transient bacterial DNA.

### Pilot clinical study results

A total of 8 participants were recruited: 6 symptomatic and 2 asymptomatic. Ethnic diversity was found among the assessed population: African (2/8), Asian (2/8), Caucasian (3/8), South American (1/8). All participants reported no current tobacco smoking habits except for one who was a former smoker. Most participants were retired, but three were university students, and one was an engineer. In terms of general medical condition, all symptomatic patients had gastroesophageal reflux disease (GERD), whereas asymptomatic individuals did not report any medical conditions.

The dental examination showed that all participants had periodontal disease, with plaque scores varying from 20 to 100%. Most cases were gingivitis (symptomatic 4/6 and asymptomatic 1/2), but 3 individuals had periodontitis (symptomatic 2/6 and asymptomatic 1/2) with Community Periodontal Index of Treatment Needs (CPITN) 3 or 4 in one or more sextants.

**Fig. 1:**
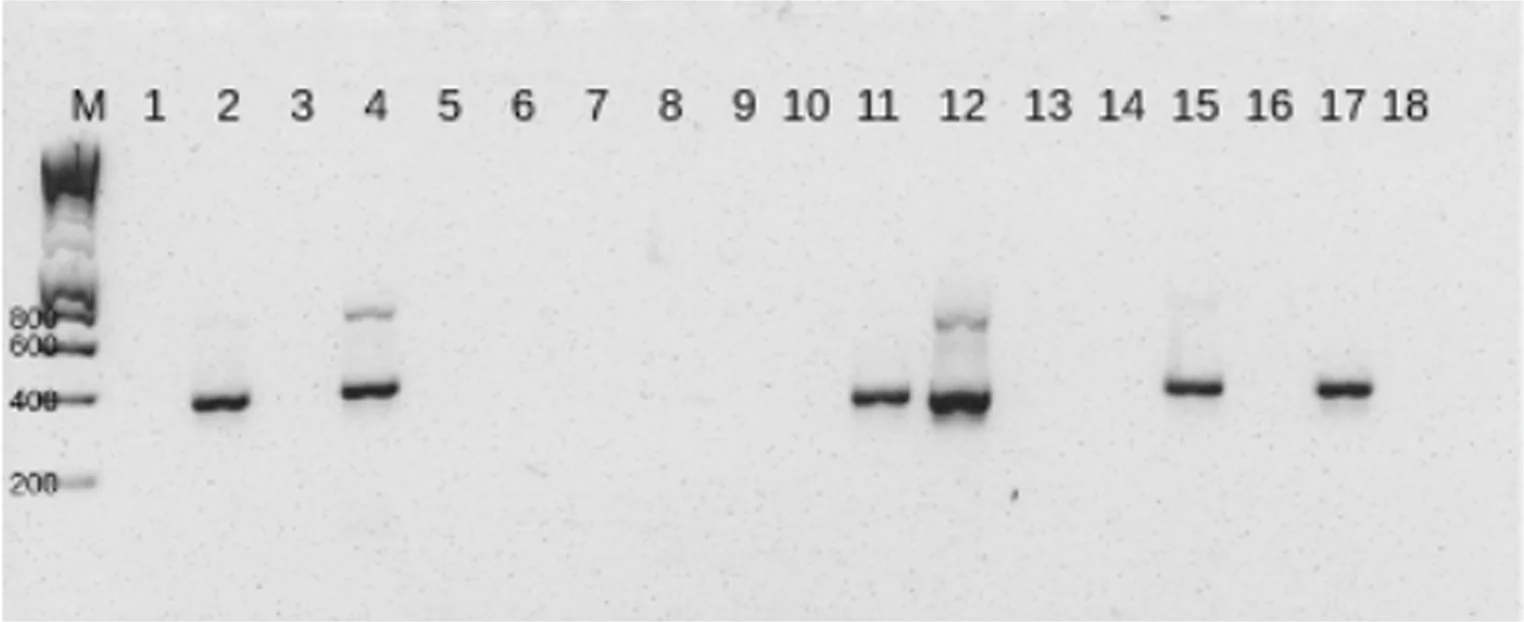
Nested PCR using 16 S primer. (M) Molecular mark − 1kB Ladder; (1) Asymptomatic Participant 1 - DP; (2) Asymptomatic Participant 1 - OM; (3) Asymptomatic Participant 2 - DP; (4) Asymptomatic Participant 2 - OM; (5) Symptomatic Participant 3 - DP; (6) Symptomatic Participant 3 - OM; (7) Symptomatic Participant 4 - DP; (8) Symptomatic Participant 4 - OM; (9) Symptomatic Participant 5 - DP; (10) Symptomatic Participant 5 - OM; (11) Symptomatic Participant 6 - DP; (12) Symptomatic Participant 6 - OM; (13) Symptomatic Participant 7 - DP; (14) Symptomatic Participant 7 - OM; (15) Symptomatic Participant 8 - DP; (16) Symptomatic Participant 8 - OM; (17) Positive *H. pylori* control; (18) Negative control. (Dental plaque – DP; Oral mucosa – OM)

**Fig. 2:**
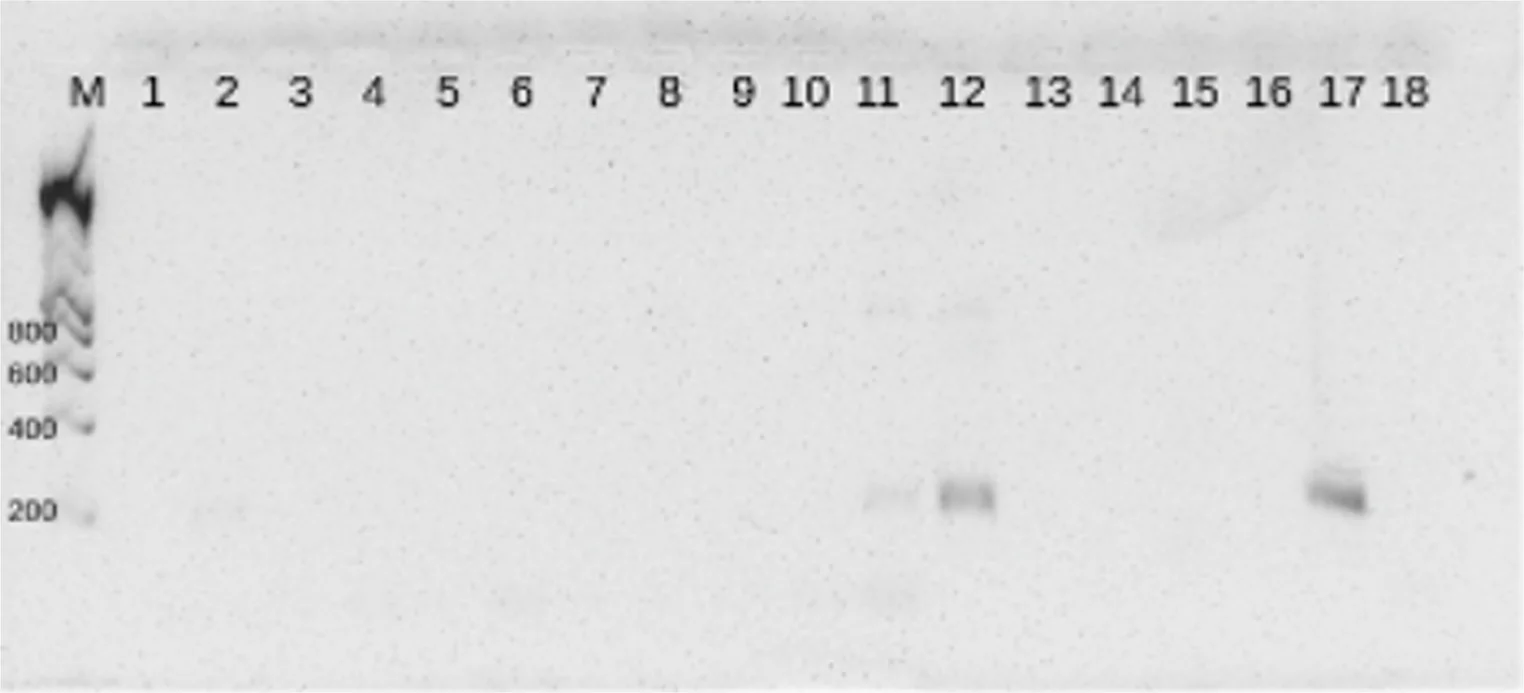
Nested PCR using ureA primer. (M) Molecular mark − 1kB Ladder; (1) Asymptomatic Participant 1 - DP; (2) Asymptomatic Participant 1 - OM; (3) Asymptomatic Participant 2 - DP; (4) Asymptomatic Participant 2 - OM; (5) Symptomatic Participant 3 - DP; (6) Symptomatic Participant 3 - OM; (7) Symptomatic Participant 4 - DP; (8) Symptomatic Participant 4 - OM; (9) Symptomatic Participant 5 - DP; (10) Symptomatic Participant 5 - OM; (11) Symptomatic Participant 6 - DP; (12) Symptomatic Participant 6 - OM; (13) Symptomatic Participant 7 - DP; (14) Symptomatic Participant 7 - OM; (15) Symptomatic Participant 8 - DP; (16) Symptomatic Participant 8 - OM; (17) Positive *H. pylori* control; (18) Negative control. (Dental plaque – DP; Oral mucosa – OM)

The gel electrophoresis results showed wells 2, 4, 11, 12, 15, and 17 positive for *H. pylori* using 16 S primer (Fig. 1) and wells 11, 12 and 17 positive using ureA primer (Fig. 2). At the participant level, *H. pylori* DNA was detected in 4 of 8 participants (50%) by nested PCR (Table 3). Among asymptomatic participants, *H. pylori* was detected in oral mucosal swabs only (participants 1 and 2, both 16 S+). Among symptomatic participants, *H. pylori* was detected in participant 6 (both plaque and mucosa, positive for both 16 S and ureA), participant 8 (plaque only, 16 S+ only), and not detected in Participants 3, 4, 5, or 7. In total, 2 of 8 plaque samples (25%) and 3 of 8 mucosal samples (37.5%) were PCR-positive (Table 2).

**Table 3:** Nested PCR results of participants

Participant number (status)	Sample	ureA primers	16 S primers
1 (asymptomatic)	Oral mucosa	-	+
	Dental plaque	-	-
2 (asymptomatic)	Oral mucosa	-	+
	Dental plaque	-	-
3 (symptomatic)	Oral mucosa	-	-
	Dental plaque	-	-
4 (symptomatic)	Oral mucosa	-	-
	Dental plaque	-	-
5 (symptomatic)	Oral mucosa	-	-
	Dental plaque	-	-
6 (symptomatic)	Oral mucosa	+	+
	Dental plaque	+	+
7 (symptomatic)	Oral mucosa	-	-
	Dental plaque	-	-
8 (symptomatic)	Oral mucosa	-	-
	Dental plaque	-	+

+ : positive detection; - : negative detection

**Fig. 3:**
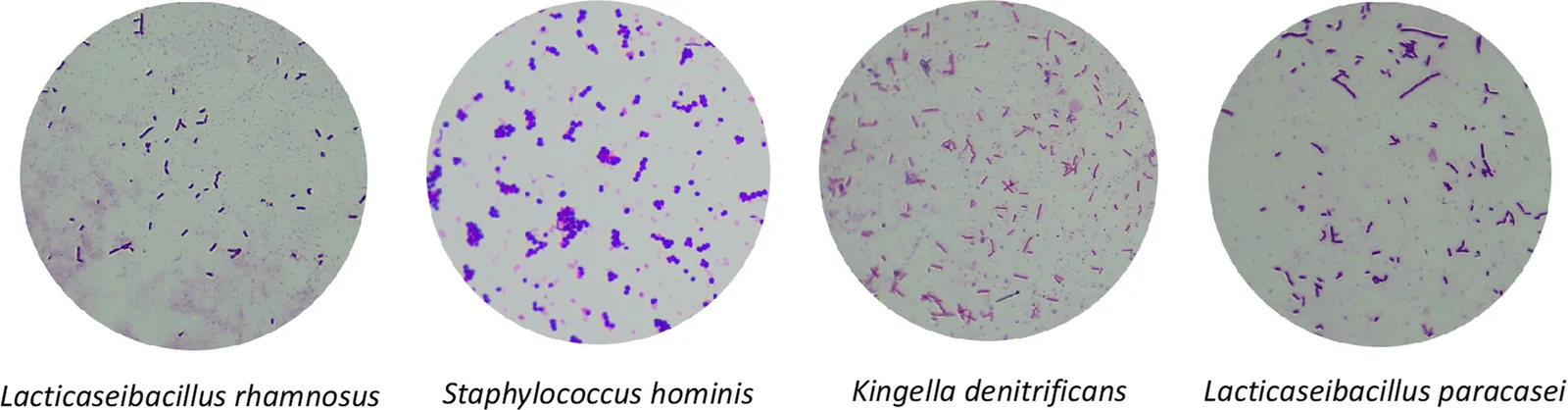
Non-*Helicobacter pylori* bacterial species isolated on selective agar plates from oral samples. Species represent organisms capable of growing on media containing vancomycin, trimethoprim, cefsulodin, and amphotericin B

In comparing the results of bacterial culture and PCR for dental plaque and oral mucosa samples, it was found that out of 8 participants, 4 (50%) detected positive oral *H. pylori* with PCR but none with culture tests (Table 4). The investigation also identified antibiotic-resistant bacterial colonies growing on the selective blood agar plates: probiotic bacteria *Lacticaseibacillus rhamnosus* and *Lacticaseibacillus paracasei*, and aerobic commensal bacteria *Staphylococcus hominis* and *Kingella denitrificans* (Fig. 3).

**Table 4:** Detection rates of *H. pylori* in dental plaque and oral mucosa in nested PCR and culture

Participants	Nested PCR		Culture	
	Hp positive in dental plaque (%)	Hp positive in oral mucosa (%)	Hp positive in dental plaque samples (%)	Hp positive in oral mucosa (%)
Asymptomatic (2)	0 (0)	2 (100)	0 (0)	0 (0)
Symptomatic (6)	2 (33)	1 (17)	0 (0)	0 (0)
Total (8)	2 (25)	3 (37.5)	0 (0)	0 (0)

Hp: *H. pylori*

## Discussion

### Synthesis of key findings from the scoping review and pilot study

This hybrid study, comprising a systematic scoping review and a pilot clinical investigation, provides an integrated understanding of the persistent methodological controversy surrounding the detection of *H. pylori* in the oral cavity. The scoping review synthesized data from over 3,000 participants across 39 studies and revealed a consistent pattern: molecular methods frequently detected *H. pylori* DNA in a wide range of oral niches, whereas successful culture of viable organisms was rare and confined to highly protected or unusual sites such as root canals or saliva treated with aggressive pre-processing methods (Table 2). The review focused on mapping the breadth and heterogeneity of available evidence on oral *H. pylori* detection rather than evaluating study validity. As the included studies encompassed a wide range of designs, populations, and laboratory methods, a unified critical appraisal approach was neither feasible nor appropriate.

The pilot clinical study reproduced this field-wide paradox. Using highly sensitive nested PCR, *H. pylori* DNA was detected in 31% of oral samples, particularly in mucosal swabs, yet no cultures yielded viable organisms despite rigorous microaerophilic techniques. Together, these findings reinforce that, while oral PCR detection is common, the biological significance and viability of oral *H. pylori* remain uncertain.

### Clarifying PCR detection patterns: insights from the review and pilot findings

The findings of this study align closely with patterns reported in the literature, where PCR-based detection of *H. pylori* in the oral cavity varies widely depending on sampling site, population characteristics, and molecular technique. Across the scoping review, detection rates ranged from 5% [34] to 90% [18]. Reviewed studies consistently demonstrated significantly higher positivity in subgingival biofilm compared with supragingival plaque. Several investigations [22, 34, 37, 48], have shown that deep periodontal pockets offer a more favourable microenvironment due to reduced oxygen tension and the presence of obligate anaerobes. The presence of periodontal pockets could also provide a more protected environment from pH changes and antimicrobial substances in saliva [54, 55]. Previous in vitro studies have suggested that interactions or co-aggregation between *H. pylori* and periodontal microorganisms such as *Fusobacterium nucleatum* and *Porphyromonas gingivalis* may enhance bacterial persistence within biofilms. However, these observations should be interpreted cautiously, particularly because well-characterised oral *H. pylori* strains are currently unavailable, and their relevance to true oral colonisation therefore remains uncertain [56]. These microecological interactions help explain why superficial supragingival plaque frequently yields negative results, whereas protected niches demonstrate higher detection rates.

Methodological variability further contributes to the heterogeneity observed across studies. Nested PCR, used in this pilot, has repeatedly shown greater sensitivity than single-step PCR, detecting *H. pylori* even when present at low biomass or in coccoid forms. This aligns with evidence from several studies summarized in Table 1, where nested PCR consistently outperformed conventional PCR, sometimes detecting DNA when single-step PCR did not. Such sensitivity, however, also increases the likelihood of detecting non-viable DNA.

In contrast to the dominant literature pattern, the present pilot study detected *H. pylori* only in oral mucosal swabs and not in supragingival plaque. This divergence is plausibly linked to sampling limitations, including the superficial nature of plaque collection and the oxygen-rich environment of supragingival plaque, which is unsuitable for a microaerophile like *H. pylori*. Meanwhile, mucosal swabs, particularly in symptomatic or reflux-prone individuals, may be susceptible to salivary coating and transient deposition of gastric *H. pylori* DNA. Recent in vitro evidence indicates that *H. pylori* can remain viable in human saliva for up to one week, particularly in the presence of other oral microorganisms [57]. Nevertheless, this transient survival does not establish that *H. pylori* can permanently colonise oral niches. Thus, the detection of *H. pylori* DNA in mucosal samples may not represent true colonisation but rather low-level, transient DNA persistence.

### Reconciling the PCR-positive/culture-negative paradox

A defining and consistent theme, both in the scoping review and in the current pilot, is the recurring PCR-positive/culture-negative discrepancy. The inability to culture *H. pylori* from PCR-positive oral samples is widely documented and reflects several biological and methodological constraints. Culture-based identification in oral samples is challenging due to the competitive overgrowth of oral microbiota that also thrive under microaerophilic conditions, often outcompeting slow-growing *H. pylori*. Additionally, culture specificity is limited because several oral taxa, including *Treponema* species, share morphological features with *H. pylori*, complicating reliable identification [56]. Therefore, previous culture reports cannot be considered definitive evidence of oral *H. pylori* colonisation, as the cultured organisms may have been *H. pylori*-related microorganisms without definitive species confirmation.

The oral environment may also promote transformation of *H. pylori* into coccoid or viable-but-non-culturable forms, which retain detectable DNA but lose culturability [45]. Evidence from culture studies in Table 2 supports this phenomenon, showing stark contrasts between high PCR positivity and near-zero viability. The present pilot findings fit this pattern: four participants were PCR-positive, yet none yielded viable *H. pylori* in culture, despite use of selective media and microaerophilic incubation. Collectively, these observations underscore that PCR positivity cannot be equated with viable colonisation, and culture negativity often reflects true biological constraints rather than methodological error.

### How the scoping review informed the pilot study design

The scoping review clearly revealed extensive variability in gene targets (including *ureA*,* glmM*,* vacA*,* cagA*, and *16 S rRNA*), PCR formats, and sampling approaches used across studies, which directly informed the design of this pilot investigation. Given the superior sensitivity reported in the literature, nested PCR was selected to maximise detection in low-biomass oral samples. The choice to sample both plaque and mucosa was grounded in comparative analyses across prior research, although the review also highlighted that superficial plaque is generally a poor reservoir for *H. pylori* due to oxygen exposure.

The scoping review’s consistent observation of PCR positivity with culture negativity was central to the justification for including both molecular and culture-based methods in this pilot. Culture remains the only method capable of confirming bacterial viability, and evaluating this methodological paradox under controlled conditions was a primary objective. The findings here mirror those of the review, providing further empirical evidence that nested PCR detects DNA even when viable organisms are not recoverable.

In retrospect, the review-supported understanding that deeper niches, such as subgingival pockets, root canals, and posterior mucosal folds, offer more favourable conditions for *H. pylori* persistence validates the negative plaque results observed in this pilot. These insights provide a clear rationale for refining sampling strategies in future research.

### Study limitations

This pilot study’s feasibility-focused design limits generalisability, and several methodological factors likely contributed to culture-negative but PCR-positive findings. Overgrowth of fast-growing oral commensals, low biomass or stressed states of *H. pylori*, and the use of standard gastric selective media may have hindered successful culture. Although samples were concentrated and incubation extended, viable-but-non-culturable cells may still have escaped detection. The small sample size and restriction to supragingival plaque, excluding the more favourable deep subgingival niche, further reduced detection sensitivity. Use of a single nested PCR target may have identified non-viable DNA, while salivary contamination may have contributed to PCR positivity in asymptomatic participants. Also, the lack of gastric biopsy data prevented strain comparison, limiting the ability to distinguish true oral colonisation from transient contamination.

### Methodological implications for future research

The integrated findings highlight several methodological priorities. First, sampling must be expanded to include subgingival plaque from deep periodontal pockets, as our scoping review consistently demonstrated higher molecular detection rates in anaerobic niches compared to supragingival plaque. Sampling may also possibly be expanded to niches such as tonsillar crypts and posterior vestibular folds.

Second, molecular detection should incorporate multi-gene panels to improve specificity and minimise false positives arising from strain heterogeneity or cross-reactivity. Several studies in Table 1 demonstrated improved detection reliability when multiple genes (e.g., *ureA*,* glmM*,* vacA*,* cagA*) were used.

Third, viability PCR methods, such as ethidium monoazide bromide (EMA) or Propidium monoazide-based PCR (PMA)-qPCR, could be used to differentiate between DNA from intact, viable cells and free DNA or dead cells [58], providing crucial insight into whether detected *H. pylori* DNA represents active colonization.

Finally, integrating a comparison of gastric-oral strains through genotyping or whole-genome sequencing will be essential to determine whether oral detection reflects true colonisation or the transient deposition of gastric DNA.

## Conclusion

This pilot study, informed by a comprehensive scoping review, demonstrated the detection of *H. pylori* DNA in oral mucosal samples using nested PCR while failing to culture viable organisms from any oral site. These findings closely mirror the overarching trend observed in the literature: PCR positivity is common across various oral niches, yet the recovery of viable *H. pylori* remains rare, inconsistent, and largely restricted to sheltered anatomical sites such as root canals.

The collective evidence suggests that the oral cavity may not consistently serve as a stable reservoir for *H. pylori*, but rather as a transient site where bacterial DNA may persist without evidence of viability. In preparation for a larger, standardised investigation, future research should prioritise sampling from deeper periodontal pockets, posterior mucosal folds, and other microaerophilic niches, while integrating gastric-oral strain comparisons to clarify whether oral detection reflects genuine colonisation or transient presence.

## Supplementary Information

Below is the link to the electronic supplementary material.

Supplementary Material 1

Supplementary Material 2

## Data Availability

No datasets were generated or analysed during the current study.

## References

[1] Martinez-Gomis J, Diouf A, Lakhssassi N, Sixou M (2006) Absence of Helicobacter pylori in the oral cavity of 10 non-dyspeptic subjects demonstrated by real-time polymerase chain reaction. Oral Microbiol Immunol 21:407–410. 10.1111/j.1399-302X.2006.00295.x 17064400

[2] Payão SL, Rasmussen LT (2016) Helicobacter pylori and its reservoirs: A correlation with the gastric infection. World J Gastrointest Pharmacol Ther 7:126–132. 10.4292/wjgpt.v7.i1.126 PMC4734945 26855818

[3] Adler I, Muino A, Aguas S, Harada L, Diaz M, Lence A, Labbrozzi M, Muino JM, Elsner B, Avagnina A, Denninghoff V (2014) Helicobacter pylori and oral pathology: relationship with the gastric infection. World J Gastroenterol 20:9922–9935. 10.3748/wjg.v20.i29.9922 PMC4123373 25110422

[4] Elad S, Zadik Y, Caton JG, Epstein JB (2019) Oral mucosal changes associated with primary diseases in other body systems. Periodontol 2000 80:28–48. 10.1111/prd.12265 31090141

[5] Anand PS, Nandakumar K and Shenoy KT (2006) Are dental plaque, poor oral hygiene, and periodontal disease associated with Helicobacter pylori infection? J Periodontol 77:692-8. 10.1902/jop.2006.050163 16584352

[6] Zhang M, Wang Z, Yu Z, Cui G, Chen Z, Wu D (2025) Decoding the host-pathogen-environment interaction: Integrated insights into Helicobacter pylori virulence and epidemiological transmission. Decoding Infect Transmission 3:100062. 10.1016/j.dcit.2025.100062

[7] Xia M, Lei L, Zhao L, Xu W, Zhang H, Li M, Hu J, Cheng R, Hu T (2025) The dynamic oral–gastric microbial axis connects oral and gastric health: current evidence and disputes. npj Biofilms Microbiomes 11:1. 10.1038/s41522-024-00623-4 PMC11696714 39747247

[8] Allaker RP, Young KA, Hardie JM, Domizio P, Meadows NJ (2002) Prevalence of helicobacter pylori at oral and gastrointestinal sites in children: evidence for possible oral-to-oral transmission. J Med Microbiol 51:312–317. 10.1099/0022-1317-51-4-312 11926736

[9] Goosen C, Theron J, Ntsala M, Maree FF, Olckers A, Botha SJ, Lastovica AJ, van der Merwe SW (2002) Evaluation of a novel heminested PCR assay based on the phosphoglucosamine mutase gene for detection of Helicobacter pylori in saliva and dental plaque. J Clin Microbiol 40:205–209. 10.1128/jcm.40.1.205-209.2002 PMC120087 11773117

[10] Shimoyama T, Horie N, Kato T, Kaneko T and Komiyama K (2000) Helicobacter pylori in oral ulcerations. J Oral Sci 42:225-9. 10.2334/josnusd.42.225 11269381

[11] Al-Ahmad A, Kürschner A, Weckesser S, Wittmer A, Rauberger H, Jakob T, Hellwig E, Kist M, Waidner B (2012) Is Helicobacter pylori resident or transient in the human oral cavity? J Med Microbiol 61:1146–1152. 10.1099/jmm.0.043893-0 22499779

[12] von Elm E, Altman DG, Egger M, Pocock SJ, Gøtzsche PC, Vandenbroucke JP (2007) Strengthening the reporting of observational studies in epidemiology (STROBE) statement: guidelines for reporting observational studies. BMJ 335:806–808. 10.1136/bmj.39335.541782.AD PMC2034723 17947786

[13] Moola S, Munn Z, Tufanaru C, Aromataris E, Sears K, Sfetc R, Currie M, Lisy K, Qureshi R, Mattis P and Mu P-F (2020) Chap. 7: Systematic Reviews of Etiology and Risk. Book title. 10.1097/XEB.0000000000000064 26262566

[14] Tricco AC, Lillie E, Zarin W, O’Brien KK, Colquhoun H, Levac D, Moher D, Peters MDJ, Horsley T, Weeks L, Hempel S, Akl EA, Chang C, McGowan J, Stewart L, Hartling L, Aldcroft A, Wilson MG, Garritty C, Lewin S, Godfrey CM, Macdonald MT, Langlois EV, Soares-Weiser K, Moriarty J, Clifford T, Tunçalp Ö, Straus SE (2018) PRISMA extension for scoping reviews (PRISMA-ScR): Checklist and Explanation. Ann Intern Med 169:467–473. 10.7326/M18-0850 30178033

[15] Yamakido H, Shigeishi H, Masumoto H, Hamada N, Kitasaki H, Kaneyasu Y, Niitani Y, Takemoto T, Sugiyama M and Ohta K (2025) Presence of oral Helicobacter pylori DNA and its association with dental hygiene in older adults. World Academy of Sciences Journal 7:1–6. 10.3892/wasj.2025.414

[16] Muharemovic A, Hadzic S, Pasic E, Jahic IM, Vukelic MG, Zoronjic A and Hodzic M (2025) Clinical evaluation and microbiological identification of helicobacter pylori in patients with oral ulcerations using the polymerase chain reaction (pcr) method. Mater Sociomed 37:48–53. 10.5455/msm.2025.37.48-53 PMC11910898 40098766

[17] Urrutia-Baca VH, Gonzalez Brosig KI, Salazar-Garza AA, Gomez-Flores R, Tamez-Guerra P and De La Garza-Ramos MA (2023) Prevalence of Oral Helicobacter pylori Infection in an Indigenous Community in Southwest Mexico. Clin Exp Gastroenterol 16:173–180. 10.2147/ceg.S424559 PMC10519207 37753185

[18] Sekar R, Murali P and Junaid M (2023) Quantification of Helicobacter pylori and its oncoproteins in the oral cavity: A cross-sectional study. Oral Dis 29:1868–1874. 10.1111/odi.14141 35092112

[19] Sruthi MA, Mani G, Ramakrishnan M, Selvaraj J (2023) Dental caries as a source of Helicobacter pylori infection in children: An RT-PCR study. Int J Paediatr Dent 33:82–88. 10.1111/ipd.13017 35771167

[20] Jara MG, Benso B, Lagos MJ, Tapia PC, Paulino MB and Silva CI (2022) PCR-detection of Helicobacter pylori from oral mucosa: A feasible early diagnostic tool. Ann Diagn Pathol 61:152022. 10.1016/j.anndiagpath.2022.152022 36058064

[21] Šeligová B, Lukáč Ľ, Bábelová M, Vávrová S and Sulo P (2020) Diagnostic reliability of nested PCR depends on the primer design and threshold abundance of Helicobacter pylori in biopsy, stool, and saliva samples. Helicobacter 25:e12680. 10.1111/hel.12680 32057175

[22] Flores-Treviño CE, Urrutia-Baca VH, Gómez-Flores R, De La Garza-Ramos MA, Sánchez-Chaparro MM, Garza-Elizondo MA (2019) Molecular detection of Helicobacter pylori based on the presence of cagA and vacA virulence genes in dental plaque from patients with periodontitis. J Dent Sci 14:163–170. 10.1016/j.jds.2019.01.010 PMC6562180 31210890

[23] Hamada M, Nomura R, Ogaya Y, Matayoshi S, Kadota T, Morita Y, Uzawa N, Nakano K (2019) Potential involvement of Helicobacter pylori from oral specimens in overweight body-mass index. Sci Rep 9:4845. 10.1038/s41598-019-41166-5 PMC6425031 30890723

[24] Kadota T, Ogaya Y, Hatakeyama R, Nomura R, Nakano K (2019) Comparison of oral flora before and after triple therapy for Helicobacter pylori eradication in patient with gastric disease. Odontology 107:261–267. 10.1007/s10266-018-0393-y 30291568

[25] Zhao Y, Gao X, Guo J, Yu D, Xiao Y, Wang H and Li Y (2019) Helicobacter pylori infection alters gastric and tongue coating microbial communities. Helicobacter 24:e12567. 10.1111/hel.12567 PMC6593728 30734438

[26] Ansari SA, Iqbal MUN, Khan TA, Kazmi SU (2018) Association of oral Helicobacter pylori with gastric complications. Life Sci 205:125–130. 10.1016/j.lfs.2018.05.026 29763614

[27] Nomura R, Ogaya Y, Matayoshi S, Morita Y and Nakano K (2018) Molecular and clinical analyses of Helicobacter pylori colonization in inflamed dental pulp. BMC Oral Health 18:64. 10.1186/s12903-018-0526-2 PMC5902987 29661188

[28] Wongphutorn P, Chomvarin C, Sripa B, Namwat W, Faksri K (2018) Detection and genotyping of Helicobacter pylori in saliva versus stool samples from asymptomatic individuals in Northeastern Thailand reveals intra-host tissue-specific H. pylori subtypes. BMC Microbiol 18:10. 10.1186/s12866-018-1150-7 PMC5789744 29378521

[29] Aksit Bıcak D, Akyuz S, Kıratlı B, Usta M, Urganci N, Alev B, Yarat A and Sahin F (2017) The investigation of Helicobacter pylori in the dental biofilm and saliva samples of children with dyspeptic complaints. BMC oral health 17:67–67. 10.1186/s12903-017-0361-x PMC5361728 28327128

[30] Castro-Muñoz LJ, González-Díaz CA, Muñoz-Escobar A, Tovar-Ayona BJ, Aguilar-Anguiano LM, Vargas-Olmos R, Sánchez-Monroy V (2017) Prevalence of Helicobacter pylori from the oral cavity of Mexican asymptomatic children under 5 years of age through PCR. Arch Oral Biol 73:55–59. 10.1016/j.archoralbio.2016.09.007 27665274

[31] Medina ML, Medina MG, Merino LA (2017) Correlation between virulence markers of Helicobacter pylori in the oral cavity and gastric biopsies. Arq Gastroenterol 54:217–221. 10.1590/s0004-2803.201700000-24 28724047

[32] Mendoza-Cantú A, Urrutia-Baca VH, Urbina-Ríos CS, De la Garza-Ramos MA, García-Martínez ME, Torre-Martínez HHH (2017) Prevalence of Helicobacter pylori vacA Genotypes and cagA Gene in Dental Plaque of Asymptomatic Mexican Children. Biomed Res Int 2017:4923640. 10.1155/2017/4923640 PMC5687131 29226140

[33] Urban J, Koszowski R, Płachetka A and Wiczkowski A (2017) An evaluation of selected oral health indicators and cariogenic bacteria titer in patients with Helicobacter pylori. Adv Clin Exp Med 26:401–407. 10.17219/acem/61907 28791813

[34] Valadan Tahbaz S, Yadegar A, Amirmozafari N, Yaghoobee S, Ehsani Ardakani MJ and Zojaji H (2017) Occurrence of Helicobacter pylori and its major virulence genotypes in dental plaque samples of patients with chronic periodontitis in Iran. Gastroenterol Hepatol Bed Bench 10:S70-s78. PMC5838184 29511475

[35] Kazanowska-Dygdała M, Duś I, Radwan-Oczko M (2016) The presence of Helicobacter pylori in oral cavities of patients with leukoplakia and oral lichen planus. J Appl Oral Sci 24:18–23. 10.1590/1678-775720150203 PMC4775005 27008253

[36] Ismail H, Morgan C, Griffiths P, Williams J and Jenkins G (2016) A Newly Developed Nested PCR Assay for the Detection of Helicobacter pylori in the Oral Cavity. J Clin Gastroenterol 50:17–22. 10.1097/mcg.0000000000000310 25811111

[37] Amiri N, Abiri R, Eyvazi M, Zolfaghari MR, Alvandi A (2015) The frequency of Helicobacter pylori in dental plaque is possibly underestimated. Arch Oral Biol 60:782–788. 10.1016/j.archoralbio.2015.02.006 25766471

[38] Bharath TS, Reddy MS, Dhanapal R, Raj Kumar NG, Neeladri Raju P, Saraswathi T (2014) Molecular detection and corelation of Helicobacter pylori in dental plaque and gastric biopsies of dyspeptic patients. J Oral Maxillofac Pathol 18:19–24. 10.4103/0973-029x.131885 PMC4065441 24959032

[39] Cai H, Li W, Shu X, Peng K, Zhang Y and Jiang M (2014) Genetic variation of Helicobacter pylori in the oral cavity and stomach detected using thymine adenine cloning in children with chronic gastritis. Pediatr Infect Dis J 33:e1-6. 10.1097/inf.0000000000000017 23989107

[40] Ogaya Y, Nomura R, Watanabe Y, Nakano K (2015) Detection of Helicobacter pylori DNA in inflamed dental pulp specimens from Japanese children and adolescents. J Med Microbiol 64:117–123. 10.1099/jmm.0.079491-0 25332373

[41] Gao J, Li Y, Wang Q, Qi C, Zhu S (2011) Correlation between distribution of Helicobacter pylori in oral cavity and chronic stomach conditions. J Huazhong Univ Sci Technolog Med Sci 31:409–412. 10.1007/s11596-011-0391-6 21671188

[42] Wichelhaus A, Brauchli L, Song Q, Adler G, Bode G (2011) Prevalence of Helicobacter pylori in the adolescent oral cavity: dependence on orthodontic therapy, oral flora and hygiene. J Orofac Orthop 72:187–195. 10.1007/s00056-011-0024-5 21744197

[43] Boyanova L, Panov V, Yordanov D, Gergova G, Mitov I (2013) Characterization of oral Helicobacter pylori strain by 4 methods. Diagn Microbiol Infect Dis 77:287–288. 10.1016/j.diagmicrobio.2013.06.030 24075629

[44] Yee KC, Wei MH, Yee HC, Everett KD, Yee HP, Hazeki-Talor N (2013) A screening trial of Helicobacter pylori-specific antigen tests in saliva to identify an oral infection. Digestion 87:163–169. 10.1159/000350432 23615458

[45] Teoman I, Ozmeriç N, Ozcan G, Alaaddinoğlu E, Dumlu S, Akyön Y, Baloş K (2007) Comparison of different methods to detect Helicobacter pylori in the dental plaque of dyspeptic patients. Clin Oral Investig 11:201–205. 10.1007/s00784-007-0104-5 17310370

[46] Loster BW, Majewski SW, Cześnikiewicz-Guzik M, Bielanski W, Pierzchalski P and Konturek SJ (2006) The relationship between the presence of Helicobacter pylori in the oral cavity and gastric in the stomach. J Physiol Pharmacol 57 Suppl 3:91–100. 17033108

[47] Cześnikiewicz-Guzik M, Karczewska E, Bielański W, Guzik TJ, Kapera P, Targosz A, Konturek SJ and Loster B (2004) Association of the presence of Helicobacter pylori in the oral cavity and in the stomach. J Physiol Pharmacol 55 Suppl 2:105 – 15. 15608365

[48] Umeda M, Kobayashi H, Takeuchi Y, Hayashi J, Morotome-Hayashi Y, Yano K, Aoki A, Ohkusa T, Ishikawa I (2003) High prevalence of Helicobacter pylori detected by PCR in the oral cavities of periodontitis patients. J Periodontol 74:129–134. 10.1902/jop.2003.74.1.129 12593608

[49] Young KA, Allaker RP and Hardie JM (2001) Morphological analysis of Helicobacter pylori from gastric biopsies and dental plaque by scanning electron microscopy. Oral Microbiol Immunol 16:178 – 81. 10.1034/j.1399-302x.2001.016003178.x 11358540

[50] Elger W, Tegtmeyer N, Rohde M, Linz B, Hirsch C, Backert S (2024) Cultivation and molecular characterization of viable Helicobacter pylori from the root canal of 170 deciduous teeth of children. Cell Commun Signal 22:578. 10.1186/s12964-024-01948-5 PMC11613870 39627817

[51] Abadi AT, Mobarez AM, Teymournejad O, Karbalaei M (2014) Concomitant Colonization of Helicobacter pylori in Dental Plaque and Gastric Biopsy. J Pathog 2014:871601. 10.1155/2014/871601 PMC4120794 25120932

[52] Wang XM, Yee KC, Hazeki-Taylor N, Li J, Fu HY, Huang ML and Zhang GY (2014) Oral Helicobacter pylori, its relationship to successful eradication of gastric H. pylori and saliva culture confirmation. J Physiol Pharmacol 65:559 – 66. 25179088

[53] Hirsch C, Tegtmeyer N, Rohde M, Rowland M, Oyarzabal OA, Backert S (2012) Live Helicobacter pylori in the root canal of endodontic-infected deciduous teeth. J Gastroenterol 47:936–940. 10.1007/s00535-012-0618-8 22722905

[54] Zaric S, Bojic B, Jankovic L, Dapcevic B, Popovic B, Cakic S, Milasin J (2009) Periodontal therapy improves gastric Helicobacter pylori eradication. J Dent Res 88:946–950. 10.1177/0022034509344559 19783805

[55] Silva MJ, Kilpatrick NM, Craig JM, Manton DJ, Leong P, Burgner D, Scurrah KJ (2019) Etiology of hypomineralized second primary molars: A prospective twin study. J Dent Res 98:77–83. 10.1177/0022034518792870 PMC6304715 30074848

[56] Agarwal S, Jithendra KD (2012) Presence of Helicobacter pylori in subgingival plaque of periodontitis patients with and without dyspepsia, detected by polymerase chain reaction and culture. J Indian Soc Periodontol 16:398–403. 10.4103/0972-124x.100919 PMC3498711 23162336

[57] Scholz KJ, Hohne A, Wittmer A, Hacker G, Hellwig E, Cieplik F, Waidner B, Al-Ahmad A (2025) Co-culture of Helicobacter pylori with oral microorganisms in human saliva. Clin Oral Investig 29:79. 10.1007/s00784-025-06160-4 PMC11757641 39849235

[58] Pan Y, Breidt F (2007) Enumeration of viable listeria monocytogenes cells by real-time PCR with Propidium Monoazide and Ethidium Monoazide in the Presence of dead cells. Appl Environ Microbiol 73:8028–8031. 10.1128/AEM.01198-07 PMC2168130 17933922

