# Supplementary material for: Oral Helicobacter pylori: a scoping review and pilot study on the PCR-culture detection paradox
Source: Clin Oral Investig. 2026 Sep 17;30(10):449. doi: 10.1007/s00784-026-07134-w (PMC13585946; doi:10.1007/s00784-026-07134-w)
Supplement: Supplementary file 1 [file 784_2026_7134_MOESM1_ESM.docx]

**Table S1. Search Terms**

| **PubMed Search (exact Boolean terms)** | ("Helicobacter pylori"[Mesh] OR "Helicobacter pylori"[tiab] OR "H. pylori"[tiab])  AND  ("oral"[tiab] OR "mouth"[tiab] OR "saliva"[tiab] OR "spit"[tiab]  OR "dental plaque"[tiab] OR "supragingival"[tiab] OR "subgingival"[tiab]  OR "oral mucosa"[tiab] OR "tongue"[tiab] OR "tongue coating"[tiab]  OR "root canal"[tiab] OR "endodontic"[tiab])  AND  ("detection"[tiab] OR "diagnosis"[tiab] OR "prevalence"[tiab]  OR "PCR"[tiab] OR "polymerase chain reaction"[tiab]  OR "qPCR"[tiab] OR "nested PCR"[tiab] OR "molecular"[tiab]  OR "culture"[tiab] OR "isolation"[tiab] OR "viable"[tiab])  NOT  ("animal"[Mesh] OR rat[tiab] OR mouse[tiab] OR mice[tiab]) |
| --- | --- |
| **Scopus** | TITLE-ABS-KEY ("Helicobacter pylori" OR "H. pylori")  AND  TITLE-ABS-KEY ("oral" OR "saliva" OR "dental plaque" OR "subgingival"  OR "oral mucosa" OR "tongue" OR "root canal")  AND  TITLE-ABS-KEY ("PCR" OR "polymerase chain reaction" OR "qPCR"  OR "nested PCR" OR "molecular" OR "culture" OR "isolation")  AND NOT TITLE-ABS-KEY ("mouse" OR "mice" OR "rat" OR "animal") |
| **Embase** | ('helicobacter pylori'/exp OR 'helicobacter pylori':ti,ab OR 'h. pylori':ti,ab)  AND  ('oral cavity'/exp OR oral:ti,ab OR saliva:ti,ab OR 'dental plaque':ti,ab  OR subgingival:ti,ab OR supragingival:ti,ab OR 'oral mucosa':ti,ab  OR tongue:ti,ab OR 'root canal':ti,ab)  AND  (detection:ti,ab OR diagnosis:ti,ab OR prevalence:ti,ab  OR pcr:ti,ab OR 'nested pcr':ti,ab OR qpcr:ti,ab  OR molecular:ti,ab OR culture:ti,ab OR isolation:ti,ab)  AND  ([humans]/lim) |
| **Web of Science** | TS = ("Helicobacter pylori" OR "H. pylori")  AND  TS = ("oral" OR "saliva" OR "dental plaque" OR "subgingival"  OR "oral mucosa" OR "tongue" OR "root canal")  AND  TS = ("PCR" OR "nested PCR" OR "qPCR" OR "molecular"  OR "culture" OR "isolation" OR "detection")  NOT TS = ("mouse" OR "mice" OR "rat" OR "animal") |
