# Supplementary material for: Oral Helicobacter pylori: a scoping review and pilot study on the PCR-culture detection paradox
Source: Clin Oral Investig. 2026 Sep 17;30(10):449. doi: 10.1007/s00784-026-07134-w (PMC13585946; doi:10.1007/s00784-026-07134-w)
Supplement: Supplementary file 2 [file 784_2026_7134_MOESM2_ESM.docx]

**Identification of studies via databases and registers**

Records identified from the following databases:

 PubMed

 Scopus

 Embase

 Web of Science

Total Records Identified: (n = 1,852)

**Identification**

Additional records identified through manual searching: 0

Records after duplicates removed: 1,276

*Reasons include: (n = 1,152)*

 Non-human /*in vitro* studies
 Reviews, conference abstracts
 Studies not involving oral samples
 Irrelevant outcomes

Records screened (title/abstract): 1,276

**Screening**

*Full-text articles excluded (n = 85), with reasons:*

Oral data could not be separated (pooled oral + gastric) = 21

No primary detection data (genotyping only) = 18

Wrong study design (<5 participants/case reports excluded except Kadota et al.) = 12

Not molecular or culture-based detection =15

Could not retrieve full text = 4

Duplicate population/overlapping data =15

Reports assessed for eligibility (Full text): 124

**Studies included in the scoping review: 39**

Molecular-only studies: 28

Culture-only studies: 7

Studies using both PCR and culture: 4

**Included**

**Figure S1.** PRISMA flow chart of the literature search and study selection
